# Identification and Protein Engineering of Galactosidases for the Conversion of Blood Type B to Blood Type O

**DOI:** 10.1002/cbic.202500072

**Published:** 2025-03-12

**Authors:** Christina Möller, Henrik Terholsen, Ole Schmöker, Thi Linh Anne Lê, Jan Wesche, Paula Schmiade, Esther Eppendorfer, Niklas Rimkus, Britta Girbardt, Dominique Böttcher, Gottfried J. Palm, Jens Hoppen, Michael Lammers, Andreas Greinacher, Konstanze Aurich, Uwe T. Bornscheuer

**Affiliations:** ^1^ Institute of Biochemistry, Department of Biotechnology and Enzyme Catalysis University Greifswald Felix-Hausdorff-Straße 4 17487 Greifswald Germany; ^2^ Institute of Biochemistry Department of Synthetic and Structural Biochemistry Felix-Hausdorff-Strasse 2 17489 Greifswald Germany; ^3^ Institute of Transfusion Medicine University Medicine Greifswald Sauerbruchstrasse 17475 Greifswald Germany

**Keywords:** Biocatalysis, GH110 family, Glycoside hydrolases, Protein engineering, Universal blood

## Abstract

The supply of blood products such as red blood cells poses a challenge due to rising demand and declining donor numbers. Careful matching of blood products of different types is required. Only type O of the blood types A, B, AB and O can be received by any patient without transfusion incompatibilities. Therefore, O‐type blood can be considered “universal blood” and is especially needed in emergency situations. In this study, we focused on the conversion of the B antigen by enzymatic deglycosylation to generate the H antigen determining O‐type blood. For this, we characterized several previously unstudied α‐1,3‐galactosidases belonging to the GH110 family. Our findings revealed that the α‐1,3‐galactosidase from *Pedobacter panaciterrae* (PpaGal) exhibits superior efficiency compared to previously described galactosidases. We further increased the activity of PpaGal by 2.5‐fold using site‐directed mutagenesis. Moreover, we solved two crystal structures of PpaGal, one in the apo‐state and another in complex with d‐galactose. The combination of our mutagenesis study with the solved crystal structures provides valuable information to guide further optimization of PpaGal or other B antigen converting enzymes paving the way for the easier production of universal blood from B‐type blood.

## Graphical abstract

We identified and characterized a galactosidase from *Pedobacter panaciterrae* (PpaGal), which is more efficient than previously characterized galactosidases from the GH110 family of *Bacteroides fragilis* in converting B‐type to O‐type blood. We further increased the activity of PpaGal by enzyme engineering and solved crystal structures with and without bound galactose.

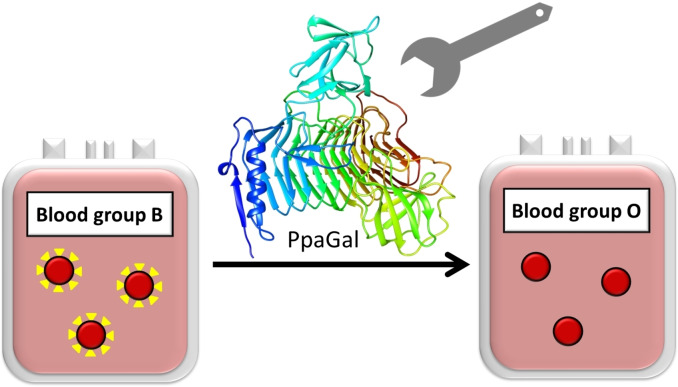

## Introduction

The transfusion of blood products plays an essential part in the modern healthcare system. According to the World Health Organization almost 120 million blood units are donated every year, however, this amount is not sufficient to counteract the required supply.^[1]^ Furthermore, the demand is constantly growing due to the demographically aging population, decreasing donor numbers since more donors are excluded for safety reasons and a rising number of blood‐intensive procedures such as organ transplantations.[^2^, ^3^] To overcome these supply challenges more donations on a regular basis are needed to ensure sufficient blood supply. However, to avoid transfusion incompatibilities, the correct matching of blood of different types must be ensured since not every patient can receive blood from every donor.

The ABO blood group system is clinically the most important system. With regard to the ABO system, only O‐type blood can be donated to any patient and can therefore be considered as “universal blood” and is especially needed in emergency situations. Therefore, it would be beneficial to find suitable enzymes to convert blood from type A and B to the O‐type. A number of enzymes were already investigated in previous studies.[^2^, ^4^, ^5^, ^6^]

**Figure 1:**
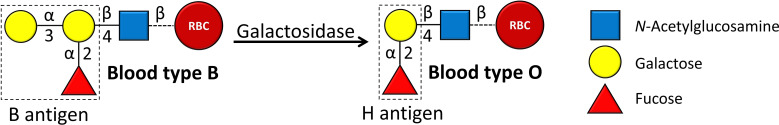
Schematic representation of enzymatic B antigen removal by a newly characterized α‐1,3‐galactosidase from *Pedobacter panaciterrae* (PpaGal) from the surface of red blood cells (RBC). Sugars are shown using the Consortium for Functional Glycomics notation.^[8]^

The B antigen that is present on red blood cells (RBCs) of type B only differs from the H antigen that defines type O by the terminal galactose unit connected by an α‐1,3‐glycosidic bond to the sub‐terminal galactose (Figure 1). Thus, removal of this additional galactose yields the H antigen of blood type O.

The concept of enzymatic removal of antigens was first demonstrated by Goldstein et al.^[7]^ For the conversion of the B antigen of blood type B to the H antigen, an α‐galactosidase from green coffee beans (*Coffea canephora*) was used. However, due to the low pH optimum (pH 3.5) a massive amount of enzyme was needed to ensure full conversion to the desired H antigen.

First clinical trials showed that the generated O‐type blood is well‐tolerated by the recipients and comparable to natural group O RBCs in terms of safety and efficacy aspects.[^9^, ^10^, ^11^] It was concluded that enzymatic conversion could indeed be used to create universal donor blood but there is a need for suitable enzymes with higher activity and improved pH optimum.^[12]^ In 2007 during a large screening of bacterial and fungal isolates, a prokaryotic family of α‐galactosidases (CAZy GH110) was identified that showed enhanced activity for the removal of the immunodominant α‐1,3‐linked galactose residues of B antigens as well as a highly restricted substrate specificity and neutral pH optimum.^[5]^ *Bacteroides fragilis* expresses two α‐galactosidases of the GH110 family which show similar behaviour in the removal of α‐1,3‐linked galactose residues from the branched blood group B antigen.[^5^, ^13^]

In this work, we identified alternative enzymes for the conversion of B‐type to O‐type blood. Given that members of the GH110B family have huge potential for the removal of galactose from B antigens, several previously unstudied members of this family were investigated. We found that the galactosidase from *Pedobacter panaciterrae* (PpaGal) is more efficient compared to the galactosidase of *Bacteroides fragilis* (BfGalB). Moreover, the enzymatic activity of PpaGal was even further improved by protein engineering and two crystal structures of PpaGal, one in complex with d‐galactose and one in the apo‐state, were solved.

## Results and Discussion

### Enzymatic Removal of B Antigens

For the identification of galactosidases with improved enzymatic activity towards the removal of the B antigen, the galactosidase from *Bacteroides fragilis* (BfGalB), a member of the GH110B family, was used as a benchmark. Based on homology, five other GH110B galactosidases with a sequence similarity between 52 % and 79 % were identified (Tables S1 and S2). The organisms harboring these galactosidases can be found in different biological niches, such as the galactosidase of *Capnocytophaga canimorsus* found in the oral flora of cats and dogs or the galactosidase of *Pedobacter panaciterrae* which was isolated from soil in South Korea.[^14^, ^15^]

All enzymes were recombinantly produced in *E. coli*, purified and biochemically characterized regarding their kinetic parameters using *p*‐nitrophenyl (*p*NP) α‐d‐galactopyranoside to determine pH optima, long‐term and thermostabilities (Table 1, Table S1, Figure S1). This model substrate enabled a convenient photometric determination of general α‐galactosidase activity.

**Table 1:** Origin of the investigated α‐1,3‐galactosidases used for the enzymatic hydrolysis using *p*‐nitrophenyl α‐d‐galactopyranoside and specific activities of purified protein.

Organism	Enzyme abbreviation	Activity [mU mg^−1^]
*Bacteroides fragilis*	BfGal	698±076
*Phocaeicola massiliensis*	PmGal	298±039
*Bacteroides pyogenes*	BpGal	377±041
*Parabacteroides johnsonii*	PjGal	381±038
*Pedobacter panaciterrae*	PpaGal	167±005
*Capnocytophaga canimorsus*	CcGal	536±010

The benchmark enzyme BfGalB showed the highest activity towards the *p*NP model substrate with a specific activity of 698±076 mU mg^−1^ compared to all other investigated galactosidases (Table 1). PpaGal was shown to have the lowest activity (167±005 mU mg^−1^) but the highest substrate affinity with a K_M_ value of 3.512±0.413 mM (Table S1). Moreover, this enzyme was also shown to have the highest residual activity after long‐term storage at 4 °C aside with the galactosidases of *Phocaeicola massiliensis* and BfGalB (all >90 %, Table S1).

**Figure 2:**
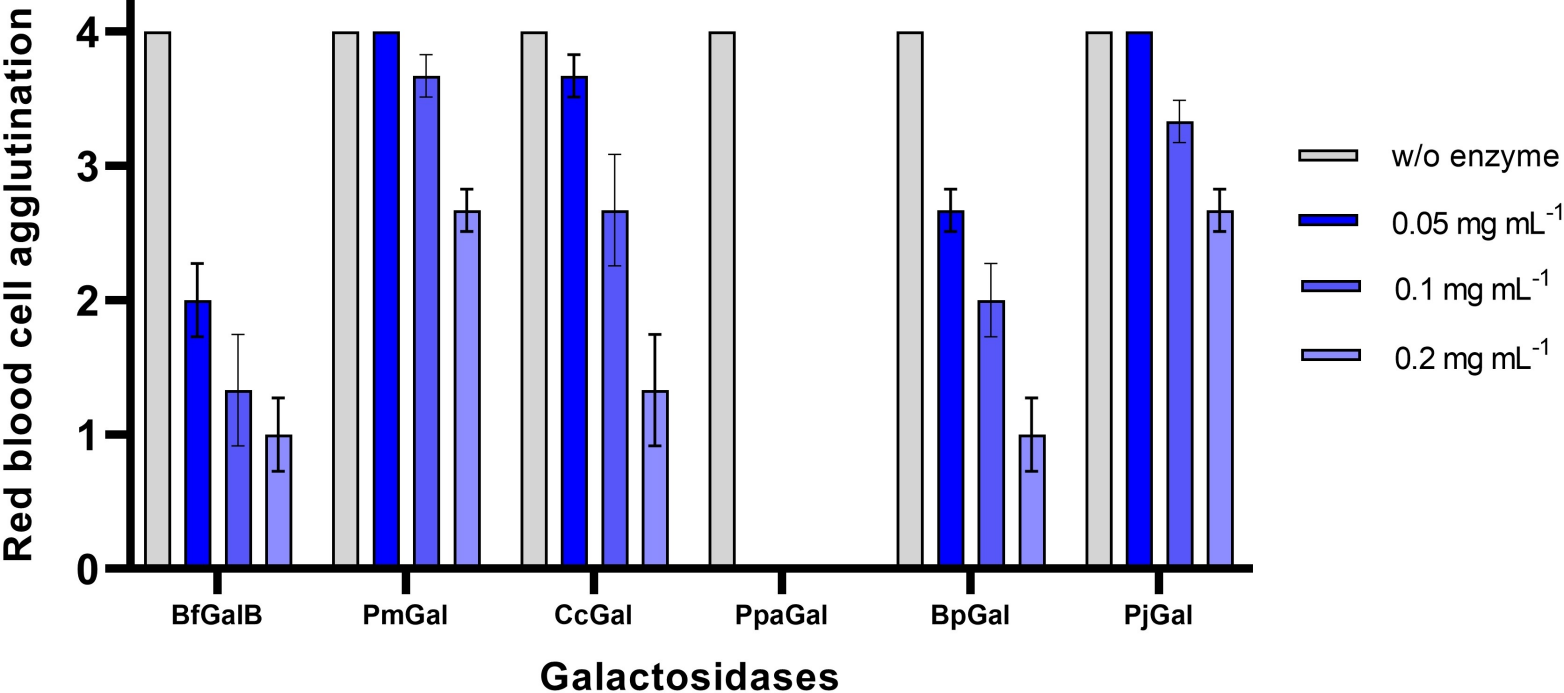
Red blood cell agglutination rates of blood type B using the galactosidases or without enzyme addition. A score of 0 indicates no agglutination with antibodies against B antigens while a score of 4 represents full agglutination. Samples were incubated for one h with 0.05 mg mL^−1^, 0.1 mg mL^−1^ or 0.2 mg mL^−1^ of the purified galactosidases.

Next, to investigate the enzyme activities toward RBCs, the enzymes were incubated with B‐type blood RBCs and agglutination tests were performed afterwards (Figure 2). If no agglutination occurs (agglutination score=0), the enzymatic removal of the B antigens was completely achieved. In contrast, if full agglutination occurs (agglutination score=4), the B antigen was not enzymatically removed. Also in these agglutination tests the galactosidase from *Pedobacter panaciterrae* was shown to be most efficient (Figure 2). Agglutination with BfGalB and the other galactosidases was still observed for all used enzyme concentrations. However, even with the lowest used enzyme concentration of PpaGal (0.05 mg mL^−1^) no agglutination with B‐antibodies occurred, which proves that the B antigen was completely removed by PpaGal.

### Protein Engineering of PpaGal

**Figure 3:**
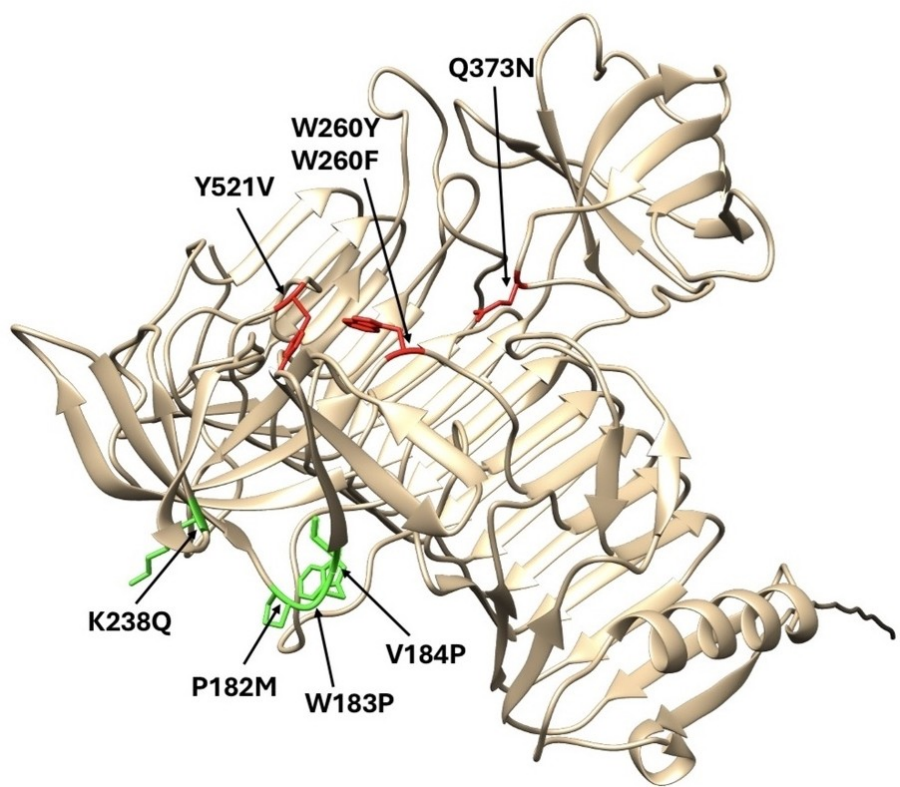
AlphaFold3 model of PpaGal. Highlighted are the mutations that were selected based on stability hotspots identified using HotSpotWizard 3.0 (green) and based on rational design (red).

Our next goal was to even further increase the activity of PpaGal in removing B antigens from RBCs and to increase the stability of the protein. For this, we investigated eight mutations that were created via site‐directed mutagenesis (Figure 3, Table S3). Four mutations were based on rational design targeting residues in close vicinity to the active site to increase the activity. Since the sugar substrate is quite polar with its number of hydroxyl groups, we followed the common approach to substitute unpolar amino acids to polar amino acids and bigger amino acids with rather smaller amino acids to create more space for substrate binding. Since PpaGal showed a melting point of 44.8 °C (Table S1), the other four mutations were based on predictions using HotSpotWizard 3.0 to increase the protein stability and are rather located on the surface of the protein.^[16]^

Six of the eight desired PpaGal mutants were successfully expressed and purified and the PpaGal variants were biochemically characterized (Table 2, Table S4). The two variants PpaGal_W260F and PpaGal_V184P were not solubly expressed and therefore not further characterized.

**Table 2:** Specific activities of the purified enzyme variants of PpaGal were assayed using *p*‐nitrophenyl α‐d‐galactopyranoside.

Mutation	Activity [mU mg^−1^]
PpaGal_wt	165±007
PpaGal_Q373N	021±004
PpaGal_W260Y	227±011
PpaGal_Y521V	004±001
PpaGal_W183P	151±027
PpaGal_K238Q	177±010
PpaGal_P182M	169±011

**Figure 4:**
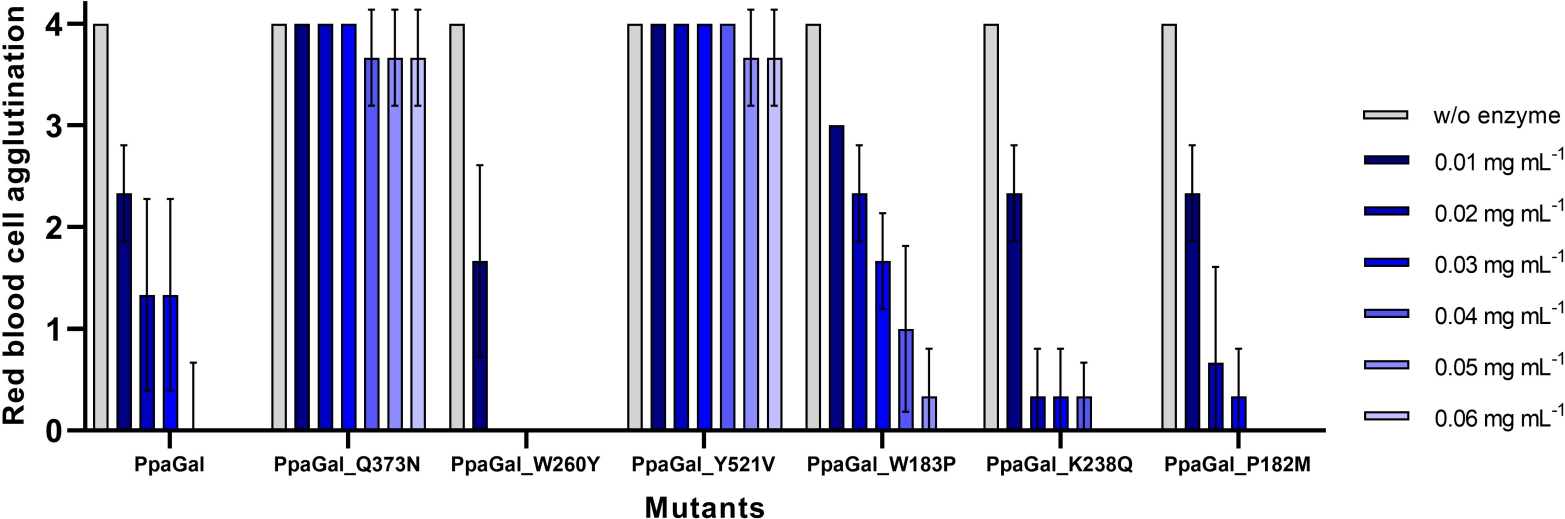
Red blood cell agglutination rates of blood type B incubated for one h with 0.01 mg mL^−1^, 0.02 mg mL^−1^, 0.03 mg mL^−1^, 0.04 mg mL^−1^, 0.05 mg mL^−1^ and 0.06 mg mL^−1^ of the expressed PpaGal mutants or without enzyme. A score of 0 indicates no agglutination with antibodies against B antigens while a score of 4 represents full agglutination.

Variants bearing the mutations W183P, K238Q and P182M, that were selected based on stability hotspots and supposed to increase the protein stability, showed slightly decreased thermostabilities in contrast to our expectations (Table S4). Moreover, similar activities compared to the wildtype (165±007 mU mg^−1^) regarding the model substrate were obtained (Table 2). Also, similar activities regarding RBCs were detected (Figure 4). This can be explained by the fact that these mutations are located on the surface of the protein, away from the active site of the protein.

All three substitutions that were based on rational protein design influenced the protein activity, regarding both, the *p*NP model substrate and the branched B antigen substrate of the surface of RBCs. While the substitution Q373N in PpaGal resulted in decreased activity, a complete activity loss was detected for PpaGal_Y521V. From these results, it can be assumed that these amino acids are important for the activity of PpaGal regarding the linear model substrate and the branched substrate.

Interestingly, the mutation W260Y increased the activity towards the *p*NP model substrate (227±011 mU mg^−1^). Also, the protein affinity for this substrate was slightly increased (Table S4). In comparison, the mutant W260F, which differs only by one hydroxyl group in comparison to the mutant W260Y, was not solubly expressed. Therefore, it can be assumed that the amino acid residue at this position plays a major role in the proteins activity and stability. The mutation W260Y also led to increased activity regarding B antigen removal of RBCs by 2.5‐fold (Figure 4). While slight agglutination was detected for RBCs treated with the wildtype of PpaGal with concentrations of 0.03 mg mL^−1^ and 0.02 mg mL^−1^, no agglutination occurred with RBCs treated with the same concentrations of PpaGal_W260Y. It can be assumed that due to the substitution of the bulky amino acid tryptophan with a smaller but still aromatic residue as tyrosine, the substrate has now greater access or more space in the active site of PpaGal.

### Crystallization of PpaGal

The results of this mutagenesis study motivated us to determine enzyme structures by X‐ray crystallography as this may help to reveal the molecular mechanisms underlying the observed impact of the different PpaGal mutations on enzyme activity. Moreover, this might enable the identification of other mutations to even further increase the activity of PpaGal or other galactosidases of the GH110 family.

**Figure 5:**
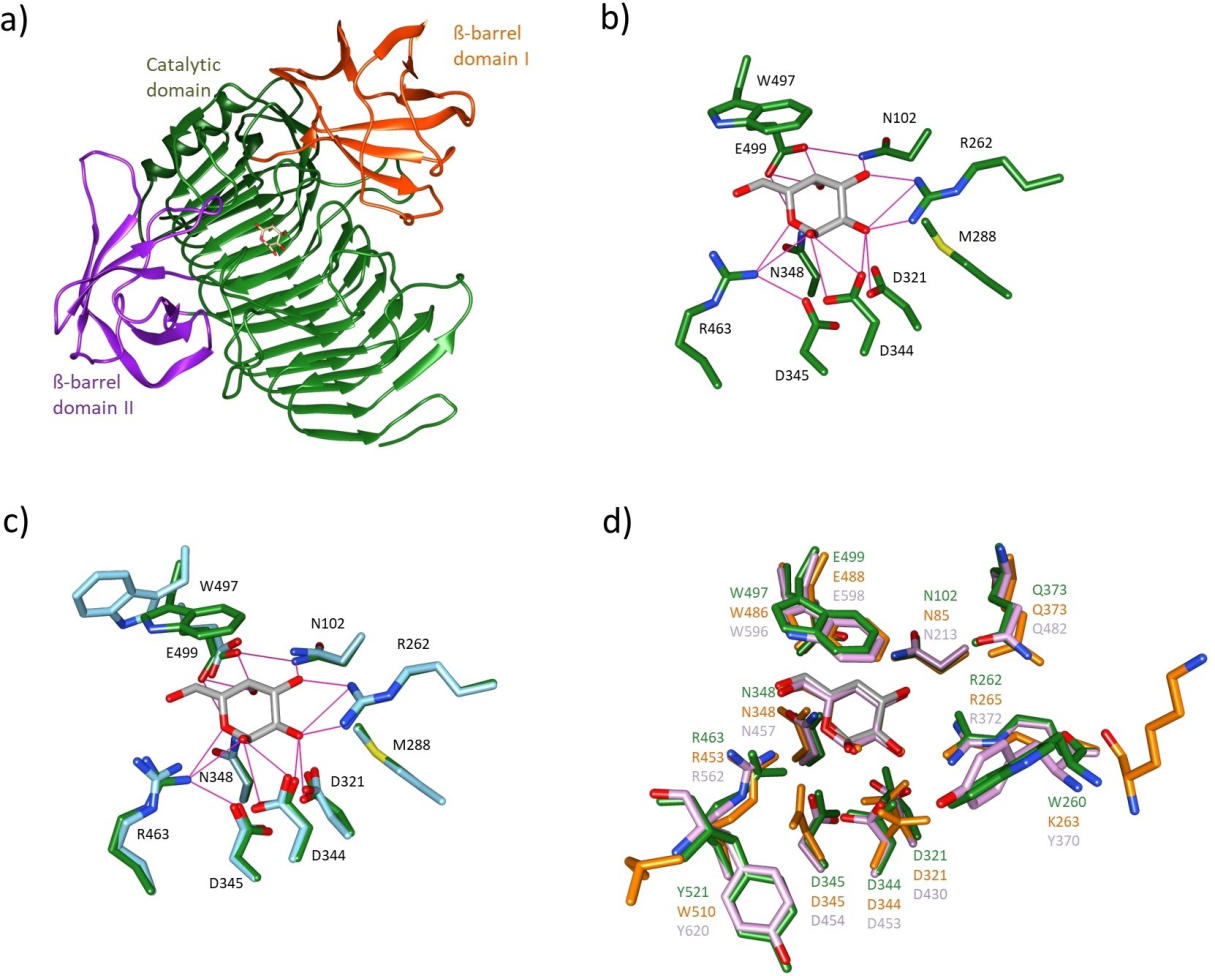
a) Crystal structure of PpaGal in complex with d‐galactose (PDB‐ID: 9I4G). Highlighted are the core structure/catalytic domain surrounded by two small β‐barrel domains (β‐barrel domain I and II). b) Relevant amino acid residues of the catalytic pocket and hydrogen bonds (pink) can be seen. c) Overlay of the two observed crystal structures of PpaGal in complex with (dark green) and without (cyan, PDB‐ID: 9I4F) d‐galactose. d) Overlay of PpaGal in complex with d‐galactose (dark green) with PdGH110B (PDB‐ID: 7JW4, orange) and AmGH110 A (PDB‐ID: 8PVS, pink). The relevant amino acids of the active site and amino acids substituted in this study based on rational design are shown. The d‐galactose in complex with PpaGal is always highlighted in dark grey.

Since crystallization approaches with PpaGal constructs containing a N‐terminal His‐tag were not successful (data not shown), a TEV‐protease‐cleavage site was introduced via site‐directed mutagenesis enabling to remove the His‐tag after purification. Indeed, after His‐tag removal, several crystals were obtained and two structures were solved (Figure 5). The structure of PpaGal in complex with galactose was refined to 2.98 Å resolution, while a resolution of 2.75 Å was obtained for the structure of the non‐complexed apo‐form of PpaGal. Analysis of the structures revealed that the core β‐helix of PpaGal is surrounded by two small β‐barrel domains (domains I and II, Figure 5a) as it is typical for enzymes of the GH110 family.[^13^, ^17^, ^18^]

The galactose is bound in the active site by an extended hydrogen bond network established with several amino acids (Figure 5b). Additionally, a stacking geometry via CH‐π‐interaction between the galactose and the tryptophan W497 was found, which often occurs in CAZymes.^[19]^

Interestingly the position of this tryptophan residue shifts if no galactose is bound (Figure 5c). Therefore, this specific tryptophan residue which is also highly conserved, seems to have a “gating function” for the substrate.

Overall, the observed crystal structures share a similar fold and similar active site residues compared to other recently solved crystal structures of the GH110 family, i. e., the galactosidase from *Pseudoalteromonas distincta* U2 A (PdGH110B) and from *Akkermansia muciniphila* (AmGH110A, Figure 5d).[^17^, ^20^] Thus, a similar inverting catalytic mechanism of the GH110 enzyme class can be assumed where the three aspartic acid residues function as the catalytic residues.[^20^, ^21^]

Interestingly, at the same position of the recently discovered α‐galactosidase of *Akkermansia muciniphila* that was found in the human gut, also a tyrosine residue is present at the position where we inserted the substitution W260Y in PpaGal that led to increased activity, even though this residue is not directly located in the active site (Figure 5d).^[17]^

These results of the mutagenesis study together with the new crystal structures present valuable data for further rational protein engineering to create more potent enzyme variants of PpaGal or other galactosidases aiming to improve the conversion of B‐type RBCs to O‐type RBCs.

## Conclusions

We identified and characterized several new galactosidases of the GH110 family that show activity in B antigen removal in order to create the universal blood type O from B‐type blood. We found that the galactosidase sourced from *Pedobacter panaciterrae* (PpaGal) proves to be more efficient at removing B antigens compared to the previously described galactosidase from *Bacteroides fragilis* (BfGalB), resulting in a reduced enzyme requirement. Furthermore, position 260 was shown to be an activity and stability hotspot in PpaGal, whereby the enzymatic activity towards B‐type RBCs was improved 2.5‐fold by the mutation W260Y. Furthermore, two crystal structures of PpaGal in its galactose free and galactose‐bound state were solved. These results provide valuable insights for the further development of efficient galactosidases for the preparation of universal blood from blood type B.

## Experimental Section

### Recombinant Protein Production and Purification

Synthetic genes for the six galactosidases to investigate B antigen removal were ordered in a pET28(a) vector (BioCat GmbH, Heidelberg, Germany). All genes contained an N‐terminal His‐tag sequence for affinity chromatography and a thrombin‐cleavage site to have the possibility to cleave the His‐tag off. The genes were codon‐optimized for recombinant expression in *E. coli*. The sequences and NCBI accession codes are listed in Table S2.

For gene expression, the pET28(a) vectors harboring the synthetic genes were transformed into *E. coli* BL21 Gold (DE3) by the heat‐shock method. For each gene, a single colony of the cells with the desired amino acid sequence was picked and used to inoculate 4 mL LB medium supplemented with 50 μg mL^−1^ kanamycin, which was grown overnight at 37 °C at 140 rpm. These starter cultures were used to inoculate the 50 mL main cultures (TB medium) in which the cells were grown until the optical density at 600 nm (OD_600_) reached approximately 0.6 ‐ 0.8. The gene expressions of the cultures were induced with a final concentration of 0.5 mM isopropyl‐β‐D‐thiogalactopyranoside (IPTG) and lasted around 20 h at 20 °C at 160 rpm. The main cultures were harvested by centrifugation (20 min, 4500 x g, 4 °C) and washed once with sodium phosphate buffer (50 mM, pH 7.5). The harvested bacteria pellets were resuspended in 4 mL washing buffer (50 mM sodium phosphate, 300 mM NaCl, 20 mM imidazole, 2 mM MgCl_2_, pH 8.0) for each gram of cell pellet. The cells were disrupted via ultrasonication with 30 % power and 50 % cycle on ice. Thereby, the sonication procedure consisted of 5 min sonication followed by a 2 min break and another 5 min of sonication. For separation of the cell debris from the supernatant, the samples were centrifuged at 10.000 × g for 30 min at 4 °C. The clarified lysates containing the desired proteins were transferred onto Ni‐IDA columns (ROTI®Garose‐His/Ni Beads, Carl Roth GmbH + Co. KG, Karlsruhe, Germany) for affinity chromatography, washed ten times with washing buffer and eluted in fractions with elution buffer (50 mM sodium phosphate, 300 mM NaCl, 2 mM MgCl_2_, 250 mM imidazole, pH 8.0). The fractions with the highest protein content were pooled and rebuffered in 50 mM sodium phosphate buffer pH 7.5 using Amicon Ultra‐15 centrifugal filter units (MWCO 10 kDa; Merck KGaA, Darmstadt, Germany). The protein solutions were stored at 4 °C until further use.

### Protein Characterization

*Protein yields*. The protein concentrations were determined by measuring the absorption at 280 nm via NanoDrop 1000 (Thermo Scientific, Waltham, MA, USA), and protein yields were calculated based on the extinction coefficients from the Expasy tool ProtParam (https://web.expasy.org/protparam/), Table S1.

*Activity tests with a model substrate*. Activity measurements were performed with *p*‐nitrophenyl α‐d‐galactopyranoside as a chromogenic substrate. The product, *p*‐nitrophenol, is colorless when protonated and becomes yellow in aqueous alkaline solutions. The production of the yellow‐colored *p*‐nitrophenolate can be measured by absorption at 405 nm. 80 μL of 50 mM sodium phosphate buffer pH 7.5 and 20 μL of a 400 μg mL^−1^ enzyme solution was pipetted into a 96‐well plate. Reactions were started by the addition of 100 μL of a 5 mM substrate solution dissolved in 50 mM sodium phosphate buffer pH 7.5 to a final reaction volume of 200 μL (final substrate concentration of 2.5 mM). Absorption at 405 nm was measured every 30 seconds over 10 min.

*pH Optima*. Activity tests were performed similarly as described above. However, instead of 80 μL of 50 mM sodium phosphate buffer pH 7.5, 80 μl of 50 mM sodium phosphate buffer with different pH values were added to the 96‐well plate (pH 6.0, 6.5, 7.0, 7.5, 8.0 or 8.5) to adjust the desired pH value. In contrast to the previous activity tests, where the substrate was dissolved in 50 mM sodium phosphate buffer pH 7.5, the substrate solutions were prepared in MilliQ water to not influence the pH value.

*Determination of K_M_ values*. K_M_ values were determined by measuring initial rates with varying substrate concentrations. First, the substrate concentrations 0 mM, 0.75 mM, 1.5 mM, 2.5 mM, 4 mM, 6 mM, 9 mM and 12 mM for CcGal and PpaGal, and 0 mM, 1.25 mM, 2.5 mM, 3.75 mM, 5.0 mM, 7.5 mM, 11 mM and 15 mM for BpGal, PmGal, PjGal and BfGalB were pipetted into a reaction plate with the same volume of 100 μl in each well. The reactions were started by the addition of 100 μl of the enzyme solutions with a concentration of 40 μg mL^−1^ of CcGal, BpGal, PmGal, PjGal, BfGalB (final concentration 20 μg mL^−1^) and 20 μg mL^−1^ of PpaGal (final concentration 10 μg mL^−1^). Calculation of K_m_ values occurred with a nonlinear regression for the fit function for the Michaelis‐Menten kinetics of the software GraphPad Prism7 (GraphPad Software, CA, USA).

*Thermostability*. The stability of the proteins was investigated by measuring melting points that serve as an indicator of the overall stability of proteins. After protein purification, the melting points of the protein samples stored in 50 mM sodium phosphate buffer pH 7.5 were investigated by using Nano differential scanning fluorimetry (Prometheus NT.48 nanoDSF, NanoTemper Technologies GmbH, Munich, Germany).

*Long‐term stability*. Besides the thermostability, the long‐term stability of a protein can be determined. For this approach, an activity test was performed as described above. A second activity test was performed after 81 days of storage at 4 °C or 20 h incubation at 25 °C and 500 rpm and residual activities were calculated.

### Blood Group Antigen Determination on Red Blood Cells

*Blood Manufacturing*. Whole blood was collected from healthy donors of blood type B according to the German guidelines for hemotherapy with written informed consent. 1 mL of the whole blood sample was centrifuged (1000 x g, 5 min) to separate blood components. 500 μL of RBCs were transferred into a new tube and 3 mL PBS buffer (PAN‐Biotech GmbH, Aidenbach, Germany) was added for washing of the RBCs. This solution was centrifuged again (1000 x g, 5 min), the supernatant was removed and the washing steps were repeated two more times. Subsequently, 200 μL of the washed RBCs of each blood sample were carefully mixed with 1400 μL PBS buffer.

*Incubation of RBCs with galactosidases*. The manufactured RBCs of blood group B were used to test the antigen removal by the different galactosidases. For B antigen removal, 200 μL were incubated with 200 μL of a 0.1, 0.2, and 0.4 mg mL^−1^ solutions of the galactosidases in 50 mM sodium phosphate buffer pH 7.4 for 1 h at room temperature (final enzyme concentration of 0.05, 0.1, and 0.2 mg mL^−1^). Afterwards, the samples were washed three times with 2 mL PBS pH 7.4 to remove the enzymes and the RBCs were resuspended in 1 mL ID‐Diluent 2 (Bio‐Rad Laboratories Inc, Hercules, CA, USA).

*Agglutination tests*. Agglutination tests with anti‐A or anti‐B antibodies (Optima Testseren, Bammental, Germany) were performed on size exclusion chromatography dextran gel cards (LISS/Coombs, Bio‐Rad, Feldkirchen, Germany) to determine the enzymatic activities of the galactosidases. 50 μL of the resuspended treated RBCs solutions were incubated with 50 μL anti‐A or anti‐B antibodies at room temperature for 15 min. Cards were centrifuged for 10 min at 910 rpm at room temperature using ID centrifuge 24 S (Bio‐Rad) and the extent of antigen removal from the RBC was evaluated by the location of the RBC in the gel of the cards. Due to complete antigen removal RBCs migrate to the bottom (Score 0, no agglutination). In the presence of the antigen, the antibody addition results in a complex formation that is therefore present on top of the column (Score 4, full agglutination). RBCs with partially removed antigens migrate in between (Score 1 to 3).

### Molecular Cloning

For point mutations of PpaGal and the introduction of a TEV‐protease‐cleavage site (ENLYFQ||S), the Q5 site‐directed mutagenesis kit (New England Biolabs, Ipswich, USA) was used. Four single point mutations were based on rational design to increase the enzyme activity and the other four point mutations were identified using HotSpotWizard 3.0. Used forward and reverse primers can be found in Table S3. The successful generation of all desired plasmids was verified via sequencing by Microsynth AG (Microsynth AG, Balgach, Switzerland).

### Crystallization and Data Collection

*His‐tag cleavage using TEV‐protease*. For crystallization approaches, a TEV‐protease cleavage site was introduced into the vector harbouring the PpaGal gene to be able to cleave the His‐tag off after purification. After expression and purification, 50 mL of a 0.8 mg mL^−1^ solution of the PpaGal construct harbouring the TEV‐protease‐cleavage site was rebuffered in 50 mM sodium phosphate buffer pH 7.5 supplemented with 1 mM DTT and treated with 1 mL of a 1.7 mg mL^−1^ solution of the TEV‐protease via dialysis (MWCO 6–8 kDa, Carl Roth GmbH + Co. KG, Karlsruhe, Germany) overnight at 4 °C.

*Size exclusion chromatography*. Before crystallization approaches, the PpaGal was further purified by size exclusion chromatography using a Superdex 75 Increase 10/300 GL column installed on an ÄKTA pure^TM^ chromatography system (Cytiva, Freiburg, Germany). As a running buffer, the measuring buffer supplemented with sodium chloride (50 mM sodium phosphate, 150 mM sodium chloride, pH 7.5) was used.

*Rebuffering and sample preparation*. 2.5 mL of the elution fraction with the highest protein fraction of the size exclusion chromatography were once rebuffered in 2 mM MES buffer supplemented with 50 mM NaCl at pH 6.5 and once in 20 mM Tris buffer at pH 7.5. In the following, the rebuffered protein samples were concentrated using Amicon Ultra‐15 centrifugal filter units (MWCO 10 kDa; Merck KGaA, Darmstadt, Germany). The protein solutions were subsequently frozen and stored at −80 °C until further use.

*Crystallization approaches*. PpaGal was crystallized at 12 mg mL^−1^ concentration using the sitting drop vapor diffusion technique. Crystallization was carried out in 96‐well CrystalQuick LP plates (Greiner Bio‐one, Frickenhausen, Germany, Cat.No. 609180) using commercially available crystallization screens. By using the CyBi‐HTCP robotic platform (CyBio AG, Jena, Germany), 0.3 μL protein and reservoir were mixed in a 1 : 1 ratio and the drops sealed with *EasySeal* adhesive foil (Greiner Bio‐One, Frickenhausen, Germany). Plates were stored at 20 °C and regularly checked for crystal formation. PpaGal crystallized in 0.2 M Lithium citrate (Carl Roth, Karlsruhe, Germany), 20 % PEG3350 (PEG‐Ion Screen, Hampton Research, Cologne, Germany) in both buffers A and B. Crystals were cryo‐protected in 0.2 M Lithium citrate (Carl Roth, Karlsruhe, Germany), 15 % PEG3350 (Sigma Aldrich, Taufkirchen, Germany), 5 % PEG400 (Carl Roth, Karlsruhe, Germany). For PpaGal in complex with its reaction product galactose, crystals were soaked for 5 min in same cryo‐condition supplemented with 0.2 M D‐galactose (Sigma Aldrich, Taufkirchen, Germany) before being flash‐frozen in liquid nitrogen and stored until data collection. Diffraction data were collected at Beamline P13, DESY Hamburg at a wavelength of 0.97626 Å.

*Structure solution and refinement*. Collected data sets for apo PpaGal and galactose‐bound PpaGal were processed using XDS package.[^22^, ^23^] All further procedures were carried out within CCP4i2 program suite (v8.0.0.19).[^24^, ^25^] The structure of PpaGal was determined by molecular replacement (MR) employing the PHASER Basic MR pipeline.^[26]^ As search template a SWISS‐MODEL generated homology‐based model was used with a query coverage of 94 %.^[27]^ Model building and inspection of electron density maps was done in COOT (v0.9.8.95).^[28]^ The structures were subsequently refined at 2.75 Å (apo PpaGal) and 2.98 Å (galactose‐bound PpaGal) in REFMAC5 including TLS refinement and local non‐crystallographic symmetry.^[29]^ Additionally, external restraints were generated with ProSMART using the *Akkermansia muciniphila* homologue AmGH110A (PDB‐ID: 8PVS) as reference.[^17^, ^30^] The quality of the protein models was validated using MolProbity.^[31]^ Complete data collection and refinement statistics are summarized in Table S5. All diffraction data and structural models are deposited in the Protein Data Bank (PDB) with respective PDB‐ID: 9I4F and 9I4G.

## Conflict of Interests

The authors declare no conflict of interest.

1

## Supporting information

As a service to our authors and readers, this journal provides supporting information supplied by the authors. Such materials are peer reviewed and may be re‐organized for online delivery, but are not copy‐edited or typeset. Technical support issues arising from supporting information (other than missing files) should be addressed to the authors.

Supporting Information

## Data Availability

The data that support the findings of this study are available in the supplementary material of this article.
