# Supplementary material for: Identification and Protein Engineering of Galactosidases for the Conversion of Blood Type B to Blood Type O
Source: Chembiochem. 2025 Mar 12;26(7):e202500072. doi: 10.1002/cbic.202500072 (PMC12002104; doi:10.1002/cbic.202500072)
Supplement: Supplementary file 1 — Supporting Information [file CBIC-26-e202500072-s001.pdf]

ChemBioChem

Supporting Information

Identification and Protein Engineering of Galactosidases for the Conversion of Blood Type B to Blood Type O

Christina Möller, Henrik Terholsen,* Ole Schmöker, Thi Linh Anne Lê, Jan Wesche, Paula Schmiade, Esther Eppendorfer, Niklas Rimkus, Britta Girbardt, Dominique Böttcher, Gottfried J. Palm, Jens Hoppen, Michael Lammers, Andreas Greinacher, Konstanze Aurich,* and Uwe T. Bornscheuer*

Supplementary Figures

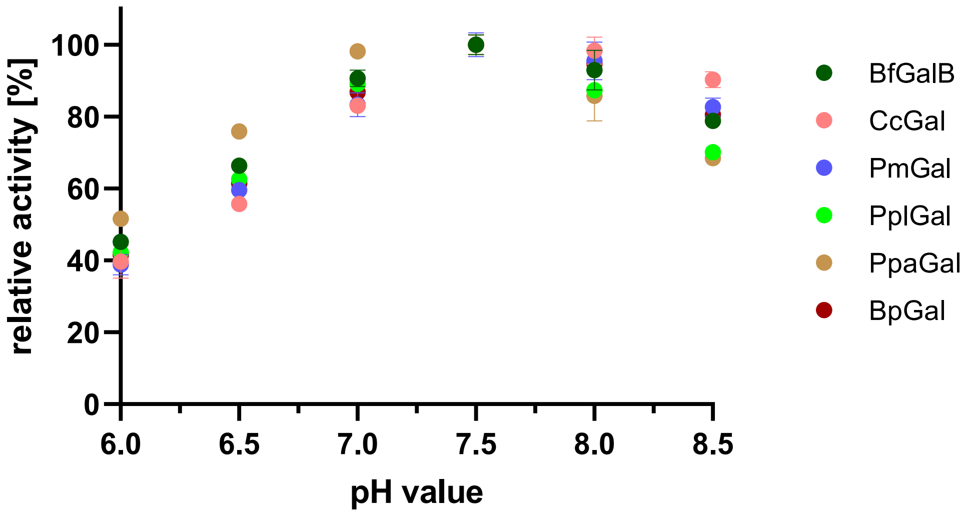

Figure S1. Determination of the pH optima of the six investigated α-galactosidases. Activity tests were performed at pH values between 6.0 and 8.5 in 50 mM sodium phosphate buffer and relative activities were calculated. n=3, mean ± standard deviation.

Supplementary Tables

Table S1. List of the original organisms of the investigated B-zymes (α-1,3-galactosidases) used for the enzymatic removal of B antigens, their sequence similarities towards the α-1,3-galactosidase of *Bacteroides fragilis*, protein yields for the recombinant expression in *E. coli* BL21 Gold (DE3) for one liter of culture volume after by IMAC purification, melting points (T_m) in 50 mM sodium phosphate buffer pH 7.5 measured with nanoDSF, residual activities after 81 days storage at 4 °C, further kinetic parameters as the catalytic constant k_{cat}, the Michaelis-Menten constant K_M and the catalytic efficiency k_{cat}/K_M values with *p*-nitrophenyl α-D-galactopyranoside as quantified at 405 nm.

Organism	**Sequence similarity (%)**	**Yield (mg L^{-1})**	**T_m (°C)**	**Residual activity (%)**	**k_{cat} (s^{-1})**	**K_M (mM)**	**k_{cat}/K_M (s^{-1} mM^{-1})**
Bacteroides fragilis	100	116.3	68.1	94.32 ± 2.50	0.753 ± 0.082	6.950 ± 0.710	0.108 ± 0.031
Phocaeicola massiliensis	79	20.9	65.3	99.31 ± 2.31	0.320 ± 0.042	13.170 ± 1.350	0.024 ± 0.009
Bacteroides pyogenes	69	95.0	63.6	88.81 ± 0.60	0.409 ± 0.045	7.667 ± 0.468	0.053 ± 0.010
Parabacteroides johnsonii	65	70.3	49.4	83.52 ± 0.70	0.418 ± 0.041	7.194 ± 0.671	0.058 ± 0.015
Pedobacter panaciterrae	58	79.0	44.8	91.25 ± 0.21	0.182 ± 0.004	3.512 ± 0.413	0.052 ± 0.011
Capnocytophaga canimorsus	52	39.9	60.6	71.90 ± 0.57	0.610 ± 0.011	4.186 ± 0.262	0.146 ± 0.019

Table S2. Amino acids sequences and NCBI codes of the synthetic genes of the investigated B-zymes containing an N-terminal His-tag and no signal peptide that were ordered in pET28a(+) vector.

BfGalB (α-1,3-galactosidase of *Bacteroides fragilis*, based on NCBI-Code: WP_010992217.1):

MGSSHHHHHHSSGLVPRGSHMASMTGGQQMGRGSEFQERVYDISQFGLKANSKKNASPVVRKAIAKIKAECRDGEKVILRFPA
GRYNFHEAGSTVREYYISNHDQDNPKKVGIALEDMKNLTIDGQGSEFVFYGRMIPVSLLRSENCVLKNFSIDFEQPHIAQVQVVE
NDPEKGITFEPAPWVDYRISKDSVFEGLGEGWVMRYSWGIAFDGKTKHVVYNTSDIGCPTKGAFEVAPRRICSPKWKDARLVPG
TVVAMRGWGRPTPGIFMSHDVNTSLLDVKVHYAEGMGLLAQLCEDITLDGFGVCLKGDNDPRYFTTQADATHFSGCKGKIVSKN
GLYEGMMDDAINVHGTYLKVIKRVDDHTLIGRYMHDQSWGFEWGRPGDDVQFVRSETMELIGKQNQITAIRPYDKGEIRGAREF
SITFKEAIDPAINEKSGFGIENLTWTPEVLFAGNTIRNNRARGTLFSTPKKTVVEDNLFDHTSGTAILLCGDCNGWFETGACRDVTI
RRNRFINALTNMFQFTNAVISIYPEIPNLKDQQKYFHGGKDGGIVIEDNEFDTFDAPILYAKSVDGLIFRNNVIKTNTEFKPFHWNKD
RFLLERVTNVKISE

PmGal (α-1,3-galactosidase of *Phocaeicola massiliensis*, based on NCBI-Code: WP_250803276.1):

MGSSHHHHHHSSGLVPRGSHMASMTGGQQMGRGSEFQKVYDISTFGLKPDTHKNASPVLQKALSKIKAECKDGEAVILRFSEG
RYDFHEKGAAVREYYISNHDQDNPKKVGIALEDMKNLTLDGQGAQFVFHGRMLPVSLLRSENCSLKNFSIDFENPHIAQVKILENT
PQEGIVFEPASWVKYRIAKDSIFEAYGEGWTLKHSWGIAFDGDTKHLVYNTSDIGCPTKGASEIAPRRIRAPHWKDARLVPGTVV
AMRGWGRPTPGIFLSHDLNTTLENIKVHYAEGMGLLAQFSENITLEKFCVCLKGEDDPRYFTTQADATHFSGCKGKITSCNGFYE
GMMDDAINVHGTYLKVVKRIDDHTLVGRYMHDQSWGFEWGRAGDEVQFVQSSTMELIGNQNKIASIRPHDKEQIDGAREFIITFD
EAIDPAVNGQSGFGIENLTWTPEVLFAGNTIRNNRARGSLFSTPRKTVVENNLFDHTSGAAILLCGDCNGWFETGACRNVIIRKNR
FVNALTNLFQFTNAVISIYPEIPDLKNQQKYFHGGPEGGIVIEDNEFDTFDAPILYAKSVDGLIFRNNVIKKNTDYKPFHSNRNRFWL
ERVTNVTIAE

BpGal (α-1,3-galactosidase of *Bacteroides pyogenes*, based on NCBI-Code: MBR8704590.1):

MGSSHHHHHHSSGLVPRGSHMASMTGGQQMGRGSEFKERVYEAARYGLKADSKRNAAPMVRKLLDKIKAEYQPGDSVVLRF
SPGRYHFHEEGATVREYYISNHDQTNPKKVGLAIEGLQHFTLDGQGAEFVFHGRMLPLSLLHSENCTLRDFSIDFANPHISQVKIV
KNEREKGITFEAAPWVKYRISKEKGFESYGEGWTGRPQTGIAFEGQTKRLVYLTSDLSIDTREAEELSAGMVYAPKWKDGRLVP
GTVVAMRTYFRPAPGIFLSHNKNTSIQRVKVHYAEGMGLLAQLCENISLDGFGVCLRSEEDPRYFTTQADATHFSACKGRIISVN
GLYEGMMDDAINVHGTYLKVVKRIDDHTLVGRYMHGQSWGFEWGRVGDRIQFVRSATMDLTGGENEVAAIEPHGTDQTEGAR
EFLIRFKQPVDERISEREGFGIENLTWTPEVVFAGNIVRNNRARGTLFSTPQQVVVENNLFDHTSGTAILLCGDCNGWYETGACR
NVLIRKNRFVNALTNMFQFTNAVISIYPEIPNLEGQQGFFHGGKDAGIIIEDNEFETFDAPILYAKSVDGLVFRRNTVRTNVDYKPFH
WNRSRFLFERVNRVQISEE

PjGal (α-1,3-galactosidase of *Parabacteroides johnsonii*, based on NCBI-Code: WP_233471270.1):

MGSSHHHHHHSSGLVPRGSHMASMTGGQQMGRGSEFKGSAYDLSSYGLNPDTGENASPLMAKALQQIAAESNSDTVRIFLPK
GRYDFYPTGSSECEYFISNHDQDNPKQVGLAFENMKNVVFDGQGSELVFHGRMLPVSLVGSDNCTLKNFSIDFANPHISQVKVL
ENDTLGGMITYEVAPWVEYEIRDSNFVAKGEGWEHVPAWGIAFEGDTKRLVYTTSDISVGSKQVAEIAPRKICAPWKNKKLIPGT
VVVFRGYGRPTPGIFMYHDTNTTLENIQVHYAEGMGLLAQMSENITLDKFSVCLRGEDDPRYFTTQADATHFSGCKGLIRSVGGL
YEGMMDDAINVHGTYLKVQKRIDDKTLVGEYMHGQSYGFEWGRPGDAVQFIDSKTMEILGKQNKVTAIEAVDKPDDHGAKQFRI
TFDKPVDPAISEAGTYGIENLEWTPEVYFADNVIRNNRARGSLFSTPKKTVVENNIFDHTSGTAILLCGDCNGWFETGACHDVLIR
NNKFINALTNQFQFTNAVISIYPEIPDLKGQKKYFHSGIVIEDNEFETFDMPILYAKSVDGLVFRNNVIKQNHDYPAFHWNNHRFFF
QRVVNAKIKDNRFEEGFDREKDIREEY

PpaGal (α-1,3-galactosidase of *Pedobacter panaciterrae*, based on NCBI-Code: NQX53349.1):

MGSSHHHHHHSSGLVPRGSHMASMTGGQQMGRGSEFNVKIYKLSAYGIKPNSGKNTTPLLTSLLKEIKSKTSDLDKVIIQFEKGR
YDFYPEGAIKREYYISNHDQDNPKTVGIGIEKFNNITLIGKGTDLMFHGRMLPLALIESSNVKIKDLNIDFEKPQITQVKIISNDTTAGN
IVFETAPWVKYKLKDSTFYNTGEGWEMQPTSGIAFENGTKHIIFNSGDIGVGTKSVSEVSPGKIMAHHWKNKKLVPGTVIAMRSW
QRPAPGIFVHKGKNISFENVKVHYAEGMGLLAQLTENIYMDGFGVCLRGKNDPRYFTTQADATHFSGCKGEIVSKNGLYEGMMD
DAINIHGTYLKITKKLDDHTVIANYMHEQSYGFDWGNIRDTVQFIQSKTMELWDAKNTIASIKPILRNSTDPIKEFRIEFTKALDPVID
PSKQDIGIENLSWTPSVVFTGNTIRNNRARGALFSTPKPTLVANNLFDHTSGCAILLCGDSNGWYETGSCRDITIRDNKFVNALTS
MYQFTSAIISIYPEIPDLTNQKKYFHSGIRILNNQFDTFDQPILYAKSVDGLVFTGNKIQTNKEYPAFHSNKKRFLFERVIGVDFSDNK
VDGKPIEML

CcGal (α-1,3-galactosidase of *Capnocytophaga canimorsus,* based on NCBI-Code: AEK23420.1):

MGSSHHHHHHSSGLVPRGSHMASMTGGQQMGRGSEFGCQKSKVENQITVADVTQKSDTVKNFSPIIQKMIGLAKAKNDESVTL
LLDKQDYYFHPEGAFVEELYISNHDQDNPKKVAFFLENMQNVTIDGGGSTLIFRGTMIPFVLKHCKNVTLKNFVIDSDEPHLRQLRI
DQVDVAKNTCIAEIQPNGKYRIEDGKLVIFGEDYQKVPNVAMAFSEDKRLAYNRPDVAFNPLKVTELKSNILKIEGWEQISKTTQG
ERFALRTYARPTPGIVIDHCKNTVVEQVKVHYANGMGLLAQLSENIHLNGFSVCLRDDKDPRYFTTQADATHFSGCKGIIRSENGL
YEGMADDAINVHGTYLKVFERVNNQTLKAKYMHHQAWGFLWGKVGDTIQFVSSETMEIIGKTNTIKSIKAIDKPSEFGAKEFEIEF
DADLHEDIKIENSVGIENLTWTPEVIFKNNIVRNNRARGALFSTPKKVICEENTFDHTHGTAILLCGDCNGWFETGACREVIIRKNKF
INALTANYQFTNAVISIYPEIPQLAKQKQFFHSGITIEDNVFETFDKPILYAKSTENILFKNNKIIYNNDFKPFHWNQYPFFFERAKGVT
LQQNDFGRPLTVEDIKVNLMPETEVKIQN

Table S3. Used primers and their sequences and annealing temperatures (T_a) for site-directed mutagenesis of PpaGal. The amino acid numbering is according to the numbering in Table S2. Positions carrying the mutation are indicated with small letters.

**Primer**	**Sequence (5' → 3')**	**T_a [°C]**
ratDesign_PpaGal_Q373N_fw	TATGCATGAAaacAGTTATGGCTTC	
ratDesign_PpaGal_Q373N_rv	TAATTGGCAATCACGG	56
ratDesign_PpaGal_W260Y_fw	CATGCGTAGTtatCAGCGTCCGG	
ratDesign_PpaGal_W260Y_rv	GCAATAACGGTACCCG	62
ratDesign_PpaGal_W260F_fw	CATGCGTAGTtttCAGCGTCCGG	
ratDesign_PpaGal_W260F_rv	GCAATAACGGTACCCG	62
ratDesign_PpaGal_Y521V_fw	GACCAGTATGgtgCAGTTCACCAGTGCAATTATTAG	
ratDesign_PpaGal_Y521V_rv	AGGGCGTTCACGAATTTATTATC	63
Stability_PpaGal_W183P_fw	AACCGCACCGccgGTGAAATATAAAC	
Stability_PpaGal_W183P_rv	TCGAACACAATATTACCG	58
Stability_PpaGal_K238Q_fw	GAGCCCGGGTcagATTATGGCCC	
Stability_PpaGal_K238Q_rv	ACTTCACTCACACTTTTGGTAC	64
Stability_PpaGal_V184P_fw	CGCACCGTGGccgAAATATAAAC	
Stability_PpaGal_V184P_rv	GTTTCGAACACAATATTACC	57
Stability_PpaGal_P182M_fw	CGAAACCGCAatgTGGGTGAAATATAAAC	
Stability_PpaGal_P182M_rv	AACACAATATTACCGGC	58
PpaGal_Tev_Introduc_fw2	tattttcagTCCGAATTCAACGTGAAAATCTACAAGCTGAGCG	34
PpaGal_Tev_Introduc_rv2	caggttttcTCCGCGACCCATTTGCTGTCCACCA	25

Table S4. List of the investigated PpaGal mutations, protein yields for the recombinant expression in *E. coli* BL21 Gold (DE3) for one liter of culture volume after by IMAC purification, melting points (T_m) in 50 mM sodium phosphate buffer pH 7.5 measured with nanoDSF, residual activities after 20 hours storage at 25°C and 500 rpm, further kinetic parameters as the catalytic constant k_{cat}, the Michaelis-Menten constant K_M and the catalytic efficiency k_{cat}/K_M values with *p*-nitrophenyl α-D-galactopyranoside as quantified at 405 nm.

Mutant	Yield (mg L^{-1})	T_m (°C)	Residual activity (%)	k_{cat} (s^{-1})	K_M (mM)	k_{cat}/K_M (s^{-1} mM^{-1})
PpaGal_wt	253.32	44.4	81.43 ± 0.78	0.18 ± 0.01	4.61 ± 0.64	0.04 ± 0.01
PpaGal_Q373N	266.64	42.8	38.36 ± 1.44	0.02 ± 0.00	4.39 ± 0.55	0.01 ± 0.00
PpaGal_W260Y	316.24	41.8	80.65 ± 0.45	0.25 ± 0.01	3.43 ± 0.34	0.07 ± 0.01
PpaGal_Y521V	121.70	-	n.d.	n.d.	n.d.	n.d.
PpaGal_W183P	57.23	36.3	38.,02± 0.30	0.16 ± 0.03	3.86 ± 0.92	0.04 ± 0.03
PpaGal_K238Q	352.88	43.7	80.55 ± 0.80	0.19 ± 0.01	5.99 ± 0.62	0.03 ± 0.01
PpaGal_P182M	373.08	42.0	77.80 ± 021	0.18 ± 0.01	5.19 ± 0.40	0.04 ± 0.01

Table S5: X-ray data collection and refinement statistics of the PpaGal in complex with D-galactose (PDB-ID: 9I4F) and in the apo-state (PDB-ID: 9I4G).

	**PpaGal apo**	**PpaGal D-galactose-bound**
**Data Collection**		
Beamline	PETRA III, EMBL c/o DESY Hamburg P13	
Wavelength (Å)	0.97626	0.97626
Space group	$P2_1$	$P2_1$
Cell dimensions		
a, b, c (Å)	107.130, 129.026, 108.296	107.011, 129.025, 108.404
$\alpha = \gamma, \beta$ (°)	90, 90.177	90, 90.129
Resolution (Å)	107.13 - 2.75 (2.8 - 2.75)	107.01 - 2.98 (3.03 - 2.98)
R_{sym}	0.167 (0.669)	0.210 (0.680)
R_{meas}	0.192 (0.785)	0.234 (0.772)
R_{pim}	0.094 (0.407)	0.103 (0.358)
$CC_{1/2}$	0.994 (0.865)	0.989 (0.834)
$\langle I/\sigma I \rangle$	6.0 (2.0)	6.4 (2.1)
Completeness (%)	96.3 (100.00)	99.4 (99.2)
Redundancy	4.0 (3.6)	5.0 (4.5)
No. of reflections	295274	298997
No. of unique reflections	73785	60001
Wilson-B-factor (Å^2)	30.94	31.60
**Refinement**		
Resoution (Å)	2.75	2.98
R_{work}/R_{free}	0.251/0.285	0.227/0.261
No. of atoms		
Protein	18556	18556
Ligand	4 (EDO)	48 (GAL)
Water	63	69
B-factors		
Protein	37.31	36.71
Ligand	55.15	29.9
Water	18.40	20.33
RMSD		
Bond lengths (Å)	0.013	0.004
Bond angles (°)	1.96	1.30
Ramachandran (%)		
Favoured	92.91	92.40
Disallowed	0.39	0.86
